# Relationships between local variability in parasite communities of the black-spotted croaker (*Protonibea diacanthus*) (Teleostei: Sciaenidae) and host population structure and seasonality

**DOI:** 10.1038/s41598-023-37428-y

**Published:** 2023-06-25

**Authors:** Megan Porter, Diane P. Barton, Mark Hearnden, Jo Randall, David A. Crook, Shokoofeh Shamsi

**Affiliations:** 1 School of Agricultural, Environmental and Veterinary Sciences, Charles Sturt University, Wagga Wagga, NSW 2678 Australia; 2 Gulbali Institute, Charles Sturt University, Wagga Wagga, NSW 2678 Australia; 3 Department of Industry, Tourism and Trade, Northern Territory Government of Australia, Darwin, NT 0801 Australia; 4 Research Institute for the Environment and Livelihoods, Charles Darwin University, Casuarina, NT 0810 Australia; 5 Arafura Timor Research Facility, Australian Institute of Marine Science, Brinkin, NT 0810 Australia; 6 Department of Primary Industries, Narrandera Fisheries Centre, Narrandera, NSW 2700 Australia

**Keywords:** Population dynamics, Parasitology

## Abstract

We evaluated spatial and temporal variability in parasite communities from the commercially important tropical marine fish the black-spotted croaker (*Protonibea diacanthus*) (Teleostei: Sciaenidae) to examine its population structure off the coast of the Northern Territory, Australia. Differences in parasite assemblage between four locations, across three seasons of the year, were used to evaluate the degree of connectivity of the sciaenid across coastal study areas. Analysis of parasite prevalence and mean intensity in these fish suggested the four sampling sites are distinct host populations. Across time, parasite assemblages at the four sites were distinct during the mid-dry (April–August) and build-up (September–November) seasons. During the wet season (December–March) there was substantial overlap in the parasite assemblages at three of the four sites indicating that fish population mixing may be occurring. Parasite assemblages at one nearshore site remained distinct across spatial and temporal scales. Our findings support the utility of parasitic organisms for elucidating the population structure of host species and reiterate the need to account for both spatial and temporal variability when performing stock discrimination analyses.

## Introduction

Understanding of fish stock structure and patterns of connectivity is critical to fisheries management, as well as being an essential baseline for spatially scaled population-level research^1–3^. Such knowledge is fundamental to understanding population dynamics and species ecology, eventually leading to a greater understanding of the resilience and potential responses of a species to exploitation, and environmental change^2^. Marine ecosystems are constantly changing, and the complexities of these systems present many challenges for defining fish populations; hence, various tools have been introduced for new methods of stock identification^1^.

The use of parasites as biological tags has long offered a powerful technique to provide information on the movement, connectivity, and biology of fishes^1,2,4–6^. The basic premise underlying the use of parasites as biological tags is that naturally occurring parasites have a discontinuous distribution from that of their host^1,4,7^. Consequently, a fish species can only become infected with certain parasites if they pass through the endemic area of that parasite^8^. Any fish harbouring a given parasite species can be marked as either coming from or having spent time in the geographical area where this parasite is endemic, and for a time after this point, these fish carry a legacy of their occupancy in that area^7,9^. The reliability of parasites as biological tags for interpreting host-fish population structure can be affected by temporal and spatial variability in parasite assemblage composition^1^. If environmental conditions change over time, either as a result of climate fluctuations, seasonality shifts, or host movements, parasite assemblages in hosts will change in response. Additionally, it is important to determine levels of local variability in fish caught within fine-scale spatial units^1^ to be able to understand the spatial relationships between different fish stocks, and also highlight the temporal influences on both parasites and fish hosts^4^.

The black-spotted croaker, *Protonibea diacanthus* (Teleostei: Sciaenidae), is commonly found in inshore and nearshore estuarine and coastal waters of northern Australia^10^. *Protonibea diacanthus* reaches sexual maturity after two years of its maximum 13-year lifespan, growing to 150 cm in length and 45 kg in weight^10^. Due to its large size and rapid growth, this species is of considerable value to recreational, traditional, and commercial fishing sectors of northern Australia, and represents one of the most commercially important fisheries of the Northern Territory^11^. A recent study based on genetic markers, otolith chemistry and parasite assemblages (see Taillebois et al.^12^), found evidence of regional population structure for *P. diacanthus*, suggesting the need for fishery management units across a spatial scale of hundreds of kilometres. Studies of fish populations at such small spatial scales are required to be able to determine levels of local variability to be able to accurately determine population structure^1^.

While the study of Taillebois et al.^12^ had wide spatial coverage, they did not examine seasonal effects on the parasite assemblage composition of the host species. *Protonibea diacanthus* has a wide distribution in the wet-dry tropics of northern Australia. In this region, the wet season is characterised by a regular appearance of a monsoonal rainfall pattern with substantially increased levels of rainfall^13^, predominately occurring over the months December to March. This typically results in large amounts of freshwater run-off from river systems into the marine environment, leading to significant changes in water temperature, salinity, and turbidity in nearshore coastal regions^14^. The dry season (occurring from April to August), in contrast, is characterised by cooler periods of little or no rainfall, with diminishing river flows^15^. The transition period from the dry to the wet season (the build-up, occurring over September to November) is when both temperature and humidity rise, with increasing regular rainfall, often short, non-monsoonal storms. The effects of these seasonal variations may have potential impacts on the physiology and demography of *P. diacanthus* and the parasites which infect them; however, this temporal variability has not been the subject of detailed research. To address this knowledge gap, we assessed parasite abundance and diversity for *P. diacanthus* populations off the northern coast of Australia to examine seasonal variation in parasite communities and their fish hosts. Based on our results, we discuss the utility of fish-parasite assemblages as biological tags in commercially important fish stock species across spatial and temporal scales.

## Material and methods

### Fish collection

**Figure 1:**
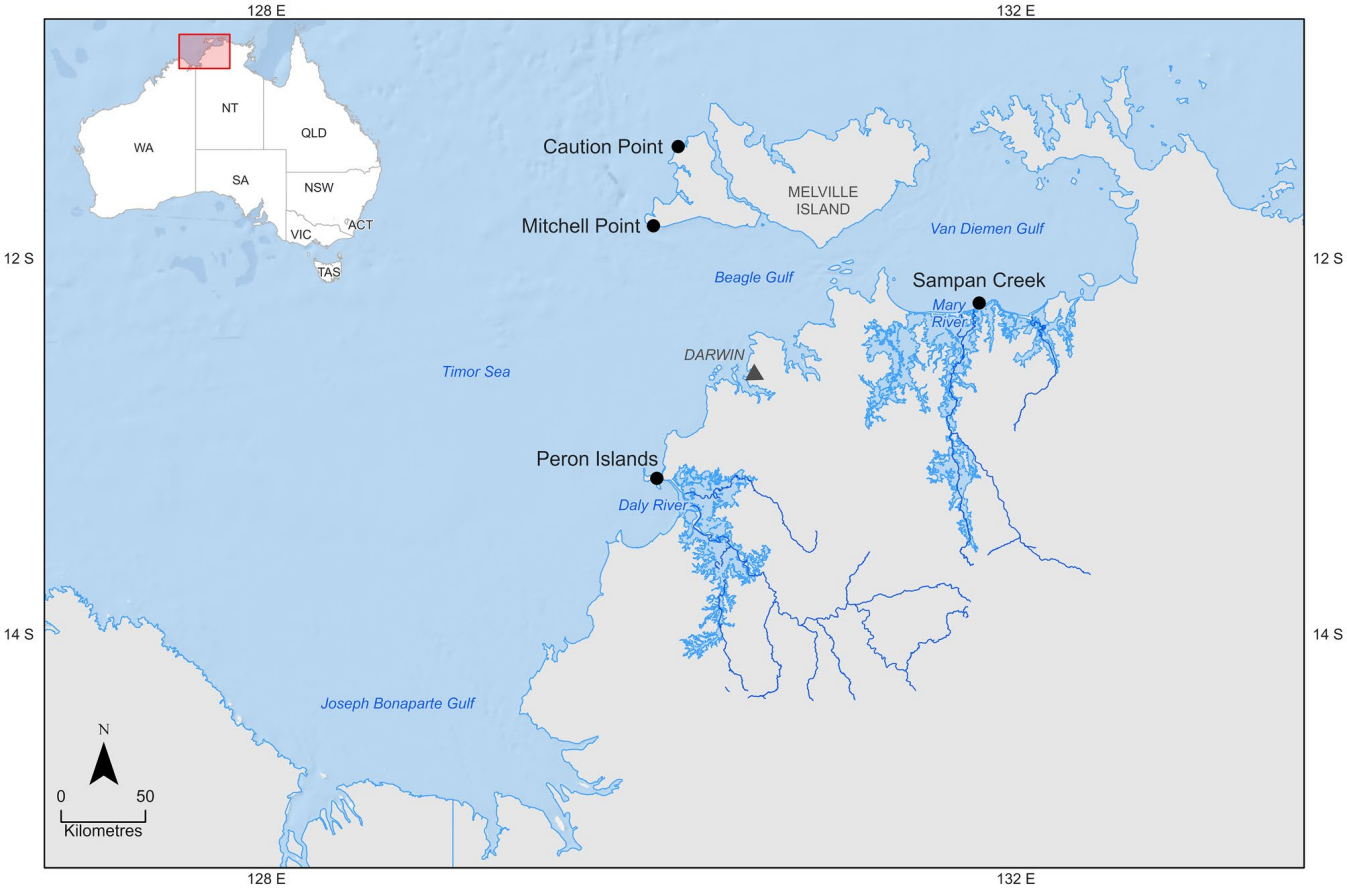
*Protonibea diacanthus* collection sites from northern Australia.

A total of 228 *P. diacanthus* were collected from four sites off the coast of the Northern Territory, Australia. The four sample sites were chosen based on proximity to freshwater outflow, with two locations offshore of the Tiwi Islands (Caution Point and Mitchell Point; the Tiwi Islands have no major river systems and, subsequently, do not have freshwater outflow), and two locations nearshore in proximity to the mouths of the Daly River and Mary River (Peron Islands and Sampan Creek, respectively) (Fig. 1). Fish were captured during three seasonal sampling periods in 2019–2021 on a seasonal event-based schedule to encompass the environmental conditions most likely to influence fish condition and physiology. These capture times were repeated over two years and included build-up (October–November 2019 and 2020), late-wet season (February–March 2020 and 2021) and mid-dry season (June–July 2019 and 2020).

Fish were collected using hook and line capture and euthanised via percussive stunning (as per Diggles^16^) (AEC approval number #A19009, Charles Darwin University). Once landed and euthanised, morphological parameters were recorded for fish total length, weight, sex, and life stage. Fish dissection was then performed on fresh samples, with both the gills and the gastrointestinal system from each fish removed, bagged, and stored frozen for later processing. All methods were performed in accordance with the relevant guidelines and regulations approved for this study (AEC approval number #A19009, Charles Darwin University).

### Parasite collection and identification

Collection of parasites followed the methodology of Taillebois et al.^12^. Parasitic cestodes and copepods were collected from the gill arch and gill filaments respectively. Other gill parasites were collected microscopically from gill wash before gill tissues were discarded. The gastrointestinal system was separated and split for the macroscopic collection of parasites. Any remaining parasites of microscopic size were collected from the gastrointestinal wash using a dissector microscope. The mesenteries and ovaries were not examined. All parasitic organisms collected were preserved in 70% ethanol and stored at room temperature for further morphological study. Parasites were identified to the lowest possible taxonomic unit using morphological characterisation (as per Taillebois et al.^12^).

### Data analysis

Summary statistics for the parasite data were compiled for each location by season of collection, with results presented for both between year variation and variation independent of year. Only parasites with a prevalence of 10% or higher (see Bush^17^) in at least one of the locations were used in the analysis. For each of the parasites deemed numerically suitable for this analysis, mean abundance (the total number of individuals of a particular parasite per sample divided by the total number of hosts examined, including uninfected hosts), and prevalence (number of hosts infected with a particular parasite divided by the number of hosts examined, expressed as a percentage) were calculated^18^. Samples were combined across the two years for analyses given variable sample sizes as a result of catch difficulties and catch vulnerabilities of *Protonibea diacanthus*. Before the samples were combined a Kruskal–Wallis H-test was performed to test for statistically significant between-year differences of parasite total abundance. Mitchell Point was excluded from this analysis as only one fish was collected in the mid-dry season of the first year.

Linear discriminant function analyses (LDFA) were conducted to provide a statistical and visual indication of the similarities of the parasite communities among samples at the regional spatial scale, and across seasons, using the MASS package in R^19,20^. Prior to analysis, data was transformed and adjusted for mean host size as described in Taillebois et al.^12^. LDFA reclassification success rates and an associated proportional chance criterion (the expected proportion of correct classification by chance alone) were calculated (as per Poulin and Kamiya^9^) for each location, and locations within the seasons. To show the separation of groups in the analyses, the first two discriminant functions for each analysis were plotted and 95% confidence ellipses shown around the centroid means of the first two discriminant functions for each group in the sample using the ellipse package in R^21^. Non-overlapping 95% confidence intervals were interpreted as the samples being significantly distinct with each other.

In accordance with relevant editorial policies, the reporting in this manuscript follows the recommendations in the ARRIVE guidelines (version 2.0).

### Ethics approval

Ethics approval for this study was provided by The Charles Darwin University (CDU) Animal Ethics Committee (AEC), approval number #A19009.

## Results

All fish were infected with at least one individual parasite and a total of 15 parasite taxa were identified from the gills and gastrointestinal system of the fish (Table 1; Supplementary Table S1a, b). Of these, two (adult isopods and unidentified digeneans) were excluded from the analysis based on a prevalence of less than 10%^17^. Collected parasites were grouped as ectoparasites (copepods and monogeneans) and endoparasites (nematodes and digeneans from the intestinal system; larval cestodes from the gill arch; and larval digenean metacercariae from the gill tissue).

**Table 1:** Prevalence and mean abundance of parasites from *Protonibea diacanthus*, across different nearshore and offshore sites of the Northern Territory, from different seasons of collection.

Season		Mid-dry								Build-up								Late-wet							
Site of collection		Caution Point		Mitchell Point		Sampan Creek		Peron Islands		Caution Point		Mitchell Point		Sampan Creek		Peron Islands		Caution Point		Mitchell Point		Sampan Creek		Peron Islands	
No. fish year 1		9		1		8		8		10		10		10		10		8		10		5		12	
No. fish year 2		10		10		12		19		7		10		10		10		8		10		9		10	
Parasite taxa		*P* (%)	*A*_*M*_	*P* (%)	*A*_*M*_	*P* (%)	*A*_*M*_	*P* (%)	*A*_*M*_	*P* (%)	*A*_*M*_	*P *(%)	*A*_*M*_	*P* (%)	*A*_*M*_	*P* (%)	*A*_*M*_	*P* (%)	*A*_*M*_	*P* (%)	*A*_*M*_	*P* (%)	*A*_*M*_	*P* (%)	*A*_*M*_
**Ectoparasites**																									
Isopod		5.26	0.05	0.00	0.00	0.00	0.00	0.00	0.00	0.00	0.00	5.00	0.05	0.00	0.00	0.00	0.00	0.00	0.00	0.00	0.00	7.14	0.07	0.00	0.00
*Caligus* sp.		10.53	0.11	0.00	0.00	17.39	0.30	66.66	1.56	17.65	0.18	0.00	0.00	5.00	0.05	10.00	0.10	0.00	0.00	0.00	0.00	7.14	0.07	22.73	0.41
*Lernanthropus paracruciatus*		78.95	2.05	100.00	6.70	82.61	3.48	55.55	2.56	82.35	3.88	95.00	5.85	95.00	12.90	95.00	4.45	93.75	7.94	70.00	2.60	71.43	5.86	90.91	2.55
Diplectanidae*		100.00	834.05	100.00	1223.00	100.00	901.09	100.00	1576.74	100.00	611.53	100.00	1328.55	100.00	195.15	100.00	113.35	100.00	2658.75	100.00	829.00	100.00	2510.71	100.00	530.91
**Endoparasites**																									
*Poecilacanstrium* sp.		100.00	27.42	100.00	21.50	100.00	46.83	100.00	36.15	100.00	28.53	95.00	21.35	100.00	39.05	100.00	40.45	100.00	26.25	100.00	22.90	100.00	46.14	95.45	19.64
Digenean metacercariae		10.53	0.16	0.00	0.00	56.52	1.78	7.41	0.19	5.88	0.06	5.00	0.05	5.00	0.15	20.00	0.65	25.00	0.31	5.00	0.10	42.86	1.07	22.73	0.23
Cucullanidae		100.00	21.89	90.00	24.40	95.65	25.26	96.30	15.85	88.24	22.47	100.00	15.35	85.00	24.00	95.00	9.35	93.75	56.44	100.00	12.95	100.00	45.64	100.00	9.82
Anisakidae		5.26	0.05	0.00	0.00	21.74	0.30	0.00	0.00	0.00	0.00	5.00	0.05	15.00	0.30	5.00	0.10	0.00	0.00	0.00	0.00	0.00	0.00	0.00	0.00
Other nematodes		26.32	0.26	10.00	0.10	8.70	0.09	29.63	0.59	0.00	0.00	10.00	0.20	20.00	0.25	25.00	0.25	0.00	0.00	0.00	0.00	21.43	0.43	13.64	0.32
*Orientodiploproctodaeum* sp.		100.00	21.53	100.00	39.10	86.96	6.43	100.00	43.74	100.00	37.00	75.00	26.10	85.00	13.05	100.00	26.35	100.00	32.25	90.00	13.75	92.86	14.29	95.45	24.36
*Stephanostomum* sp.		89.47	14.79	100.00	33.30	86.96	25.43	85.19	10.74	100.00	18.12	95.00	24.90	85.00	10.20	80.00	5.45	81.25	30.88	85.00	11.30	100.00	42.86	68.18	3.41
*Pleorchis* sp.		10.53	0.11	80.00	5.30	0.00	0.00	7.41	0.22	17.65	0.47	45.00	3.50	0.00	0.00	20.00	0.30	12.50	0.25	25.00	0.50	0.00	0.00	13.64	0.23
Hemiuridae		0.00	0.00	0.00	0.00	17.39	0.17	18.52	0.37	5.88	0.06	0.00	0.00	0.00	0.00	0.00	0.00	6.25	0.19	10.00	0.15	14.29	0.57	27.27	0.41
Opecoelidae		5.26	0.05	0.00	0.00	21.74	0.30	22.22	0.52	5.88	0.06	25.00	0.30	5.00	0.10	10.00	0.10	0.00	0.00	0.00	0.00	14.29	0.14	22.73	0.50
Unidentified. Digenean		0.00	0.00	0.00	0.00	0.00	0.00	0.00	0.00	5.88	0.06	0.00	0.00	0.00	0.00	0.00	0.00	0.00	0.00	0.00	0.00	0.00	0.00	0.00	0.00

*A*_*M*_ mean abundance, *P* prevalence of infection, expressed as a percentage.

*Diplectanidae taxa have been described by Porter et al.^31^ as two new species *Diplectanum timorcanthus* n. sp. and *Diplectanum diacanthi* n. sp. from *Protonibea diacanthus*. As per Mackenzie and Abaunza^8^ parasitic organisms need to be easily identifiable and given the two species of *Diplectanum* are biologically similar these organisms have been combined for the analyses in the present study.

The distribution and abundance of the parasite fauna of *P. diacanthus* varied across the sampling locations (Table 1). Of the ectoparasites, the diplectanid monogeneans were the most prevalent, with all fish infected with these parasites. The ectoparasite with the next highest prevalence was the copepod *Lernanthropus paracruciatus*, demonstrating a much higher prevalence and mean abundance than the copepod *Caligus* sp. which, unlike *L. paracruciatus*, was not recorded at all sampling sites. The larval cestode, *Poecilanstrium* sp., occurred in high numbers, with a minimum prevalence of 95% recorded at Mitchell Point during the build-up season. Of the adult nematodes, the Cucullanidae were present in all locations, whilst the Anisakidae were found less commonly. The adult digeneans *Orientodiploproctodaeum* sp. and *Stephanostomum* sp. were most prevalent across all locations, with *Orientodiploproctodaeum* sp. being most abundant, except at Sampan Creek which had a higher abundance of *Stephanostomum* sp. The remaining digeneans from the families Hemiuridae and Opecoelidae, were most prevalent in nearshore locations.

**Figure 2:**
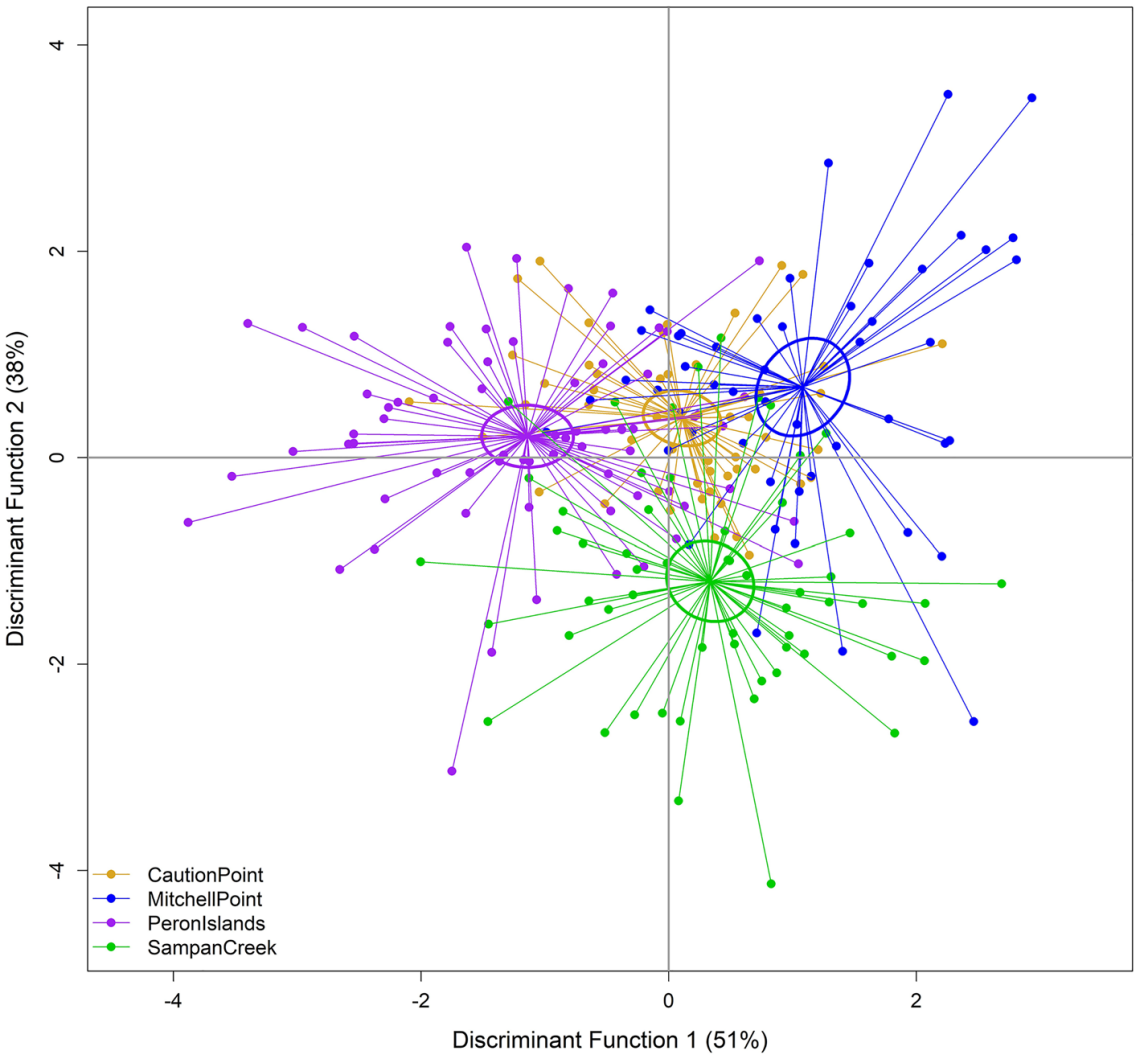
Linear discriminate functional analysis (LDFA) of parasite assemblages of all fish from all locations, independent of season. (Legend: CP Caution Point; MP Mitchell Point; PI Peron Islands; SC Sampan Creek).

The Kruskal–Wallis H-test justified the pooling of samples across the years with no significant difference found for the between-year analysis of parasite total abundance (*H*(1) = 0.70, *P* = 0.40). Independent of season, the LDFA showed the four collection locations to be significantly different from each other based on parasite assemblage (Table 2; Fig. 2). The LDFA successfully reclassified 54% of fish back to their collection location in comparison to the proportional chance criterion of 25% (Supplementary Table S2). Reclassification to individual locations was higher for the inshore locations (61% for Peron Islands and 65% for Sampan Creek) compared to the offshore locations (40% for Mitchell Point and 48% for Caution Point). Peron Islands was separated from the other three locations based on the first linear discriminant factor (LD1), which was driven primarily by higher levels of infection with *Caligus* sp. and Hemiurid digeneans. Conversely, Sampan Creek was separated from the other three locations based on the second linear discriminant factor (LD2), which was driven by higher levels of infection with Anisakidae and the digenean metacercariae, and an absence of infection with *Pleorchis* sp.

**Table 2:** MANOVA statistics from various linear discriminant functional analysis.

Analysis	Pillai's trace statistic	F statistic	Df	*p* value	Reference figure
All locations with seasons combined	0.8819	6.8543	3, 224	< 0.001	Fig. 2
All locations during mid-dry	1.5221	5.1493	3, 75	< 0.001	Fig. 3(A)
All locations during build-up	1.0786	2.9937	3, 73	< 0.001	Fig. 3(B)
All locations during late-wet	1.1121	2.8962	3, 68	< 0.001	Fig. 3(C)
All seasons at Caution Point	0.8760	3.1954	2, 49	< 0.001	Fig. 4(A)
All seasons at Mitchell Point	0.8192	2.7058	2, 47	< 0.001	Fig. 4(B)
All seasons at Sampan Creek	1.0437	4.0020	2, 54	< 0.001	Fig. 4(C)
All seasons at Peron Islands	0.8354	3.3477	2, 66	< 0.001	Fig. 4(D)

**Figure 3:**
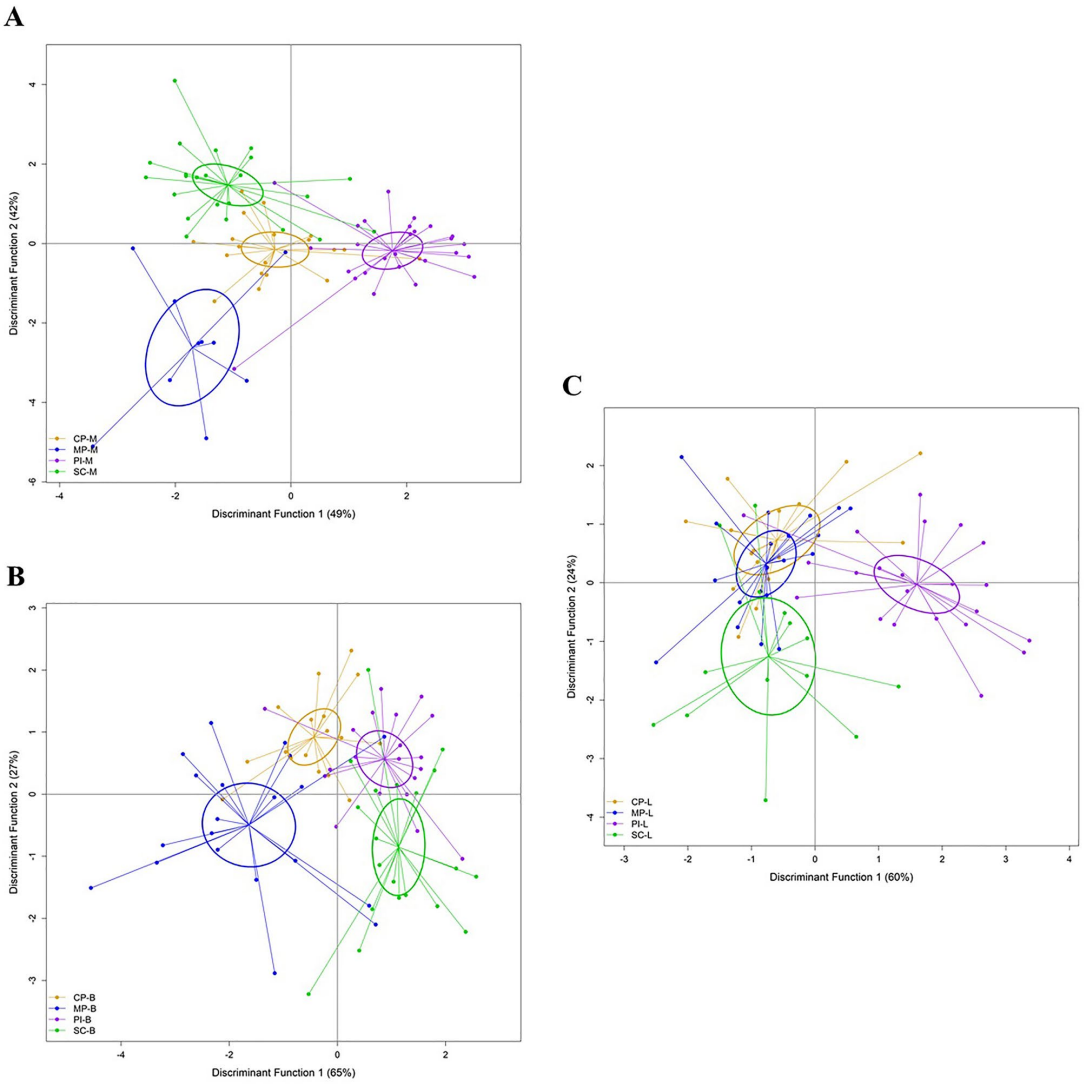
Linear discriminate functional analysis (LDFA) of parasite assemblages of all fish from each location, grouped by season. (**A**) represents samples from the mid-dry season, (**B**) the build-up season, and (**C**) the late-wet season. (Legend: B build-up; L late-wet; M mid-dry; CP Caution Point; MP Mitchell Point; PI Peron Islands; SC Sampan Creek).

Separation of the locations within seasons, showed similar patterns for the build-up and the mid-dry, with all locations significantly separated from each other (Table 2; Fig. 3). The LDFA successfully reclassified 48% (build-up) to 71% (mid-dry) of fish to their collection location, compared to the proportional chance criterion of around 25% (Supplementary Tables S3–S5). However, the results for the late-wet showed overlap in the parasite assemblages for the two offshore locations. During the late-wet the parasite assemblages of the nearshore locations had no overlap with other sites, however Sampan Creek was more similar to the offshore sites than to Peron Islands, which was significantly different (Fig. 3C). Peron Islands was distinct from the other three locations based on LD1, which was driven primarily by the much higher levels of infection with *Caligus* sp.

**Figure 4:**
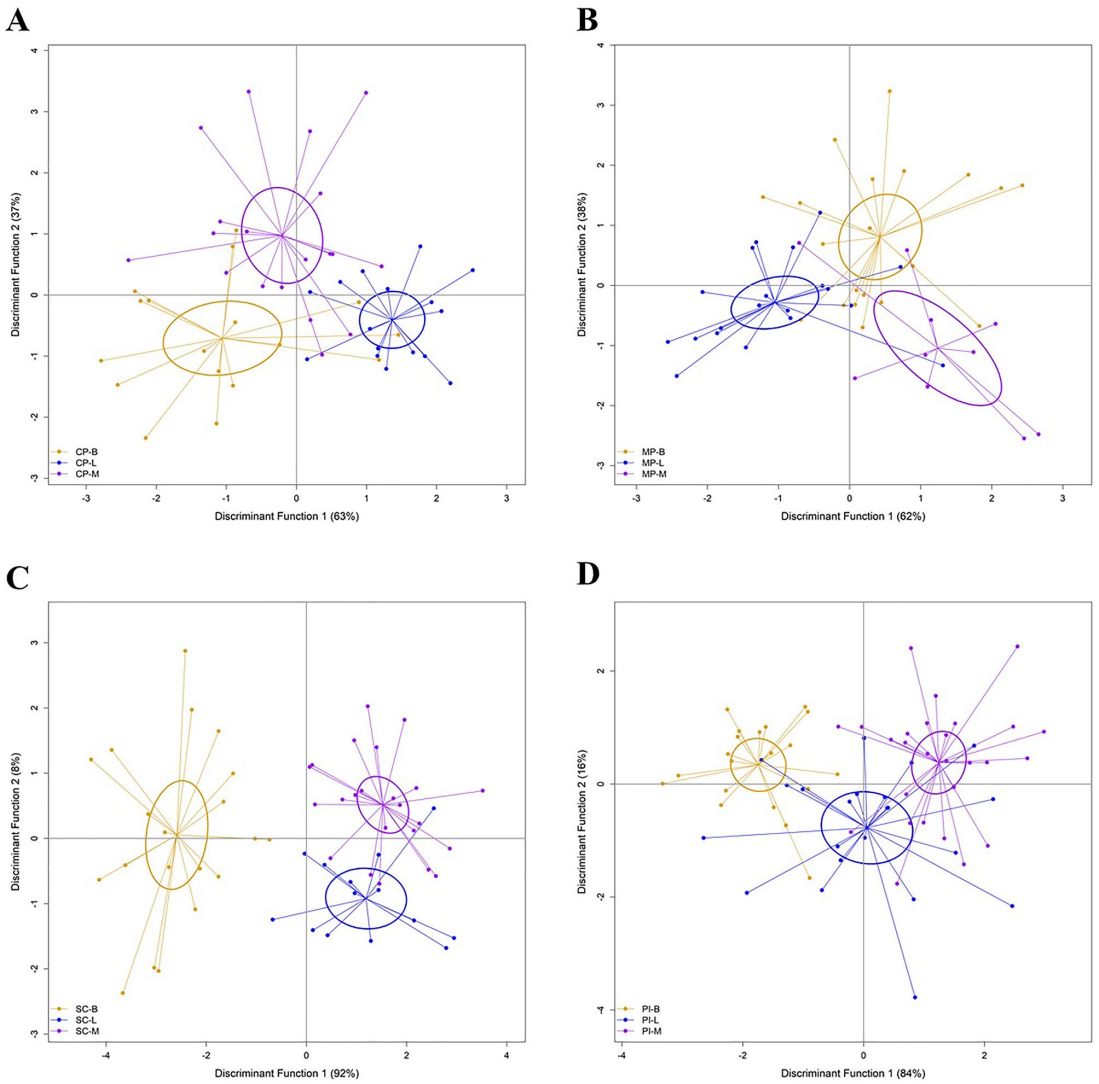
Linear discriminate functional analysis (LDFA) of parasite assemblages of all fish from each season, grouped by location. (**A**) represents samples from Caution Point, (**B**) from Mitchell Point, (**C**) from Sampan Creek, and (**D**) from Peron Islands. **(**Legend: B build-up; L late-wet; M mid-dry; CP Caution Point; MP Mitchell Point; PI Peron Islands; SC Sampan Creek).

Separation of season by location showed significant seasonal variations in parasite assemblage composition within each collection location (Table 2; Fig. 4). Within each season, the LDFA had higher classification success at the two nearshore locations (> 60%) than the offshore locations (< 60%) (Supplementary Tables S6–S9). Differences in levels of infection with *Caligus* sp., Hemiurid digeneans and ‘Other nematodes’ were the main drivers of the differences between season within each location.

## Discussion

Parasite assemblages are dynamic communities which can vary within an individual host species based on both temporal and spatial factors. Despite this, parasites can be ideal indicators of host population variance and movements, however, the type of parasites, or parasite community, utilised as biological/ecological tags, must be carefully considered. Utilising the entire parasite community of a host allows for more thorough and higher-level conclusions on host population structuring to be drawn^4,12^. Determination of the extent of temporal stability of a parasite community across different seasons, such as those in this study, is needed to evaluate the suitability of parasites in stock structure studies^1^. It is evident from this study that there is temporal variability in parasite assemblage composition among seasons that has the potential to confound stock structure assessment if not adequately considered. Despite this temporal variability, the results of the current study are consistent with the overall findings of Taillebois et al.^12^, who reported populations of *P. diacanthus* off northern Australia to be highly complex and spatially distinct. However, we also found some evidence of mixing of regional populations during the wet season, with overlap in parasite assemblage composition evident between the two offshore sites (Caution Point and Mitchell Point), suggesting that *P. diacanthus* may disperse more widely during the wet season than other times of year.

The climatic conditions of the tropical wet season are significantly different to those of the dry and build-up seasons where many environmental factors remain unchanged over a long duration. During the dry season, river flow regimes are characterised by varying degrees of flow cessation, or intermittency^15^, with subsequent changes in salinity levels and water temperatures^13^ compared to the wet season when rivers are in spate and combined with strong tidal currents create high turbidity levels in nearshore waters^14^. The fish sampled from Peron Islands have a parasite assemblage that is independent of the other populations, irrespective of the time of year. Although Taillebois et al.^12^ identified an exponential decay in the similarity of parasite assemblages as the distance between host populations increased (see Poulin & Kamiya^9^), the distance between Sampan Creek and the offshore locations is greater (approximately 240 km) compared to Peron Islands (approximately 120 km). Thus, distance alone is not sufficient to explain the distinctiveness of the parasite assemblages at Peron Islands. Previous research suggests that tidal flows and ocean currents might influence biotic assemblage composition, including parasites, in this region. Condie^22^ showed that tidal flows from the Mary River (Sampan Creek) tracked north-west towards the Tiwi Islands into the Timor Sea, whereas the tidal flows from the Daly River (Peron Islands) tracked west across the Joseph Bonaparte Gulf (JBG). Therefore, the similarity in parasite assemblages between fish populations from Sampan Creek, Mitchell Point and Caution Point in the late wet season may be more influenced by tidal flows. Additionally, the shallow water environment of the JBG with its warm, low salinity waters and increases in sediment discharge and resuspension could influence the composition of benthic organisms in this area of water, with previous surveys confirming that biota in the inner areas of JBG differ from the biota from the adjacent Timor Sea^23,24^.

The main parasite taxa driving the results of the analyses in our study were the ectoparasitic copepod *Caligus* sp. and the two digeneans Hemiuridae and *Pleorchis* sp., a finding which confirmed the need to study different components of parasite communities or assemblages of hosts, when conducting population studies^24^. Ectoparasites, such as copepods, and endoparasites, such as digeneans, have different routes of transmission to hosts, and, thus, reflect different aspects of the host’s biology and ecology. Ectoparasites, generally, have a direct lifecycle and, due to their positioning in the gills, are directly affected by environmental shifts and fish behaviours, making them ideal indicators of short-term influences^25^. Endoparasites, on the other hand, tend to utilise trophic transmission in aquatic systems, and are indirectly mediated by changes in the abundance and distribution of their hosts (especially any intermediate hosts). Therefore, any changes seen during different seasons of the year will often be indirect and reflect conditions experienced by earlier hosts than *P. diacanthus*, thus providing information on a longer time scale. Depending on the type of parasite, the local host environment can buffer parasites from environmental change to some degree. However, as host condition (including immunology and physiology) and host behaviour and distribution (which affects availability for infection) becomes affected by environmental change, these changes to the host can indirectly influence parasites^26,27^.

While our study has provided information on spatial and temporal variation in the parasite assemblages of *P. diacanthus*, it is important to consider the limitations of this study. Despite substantial sampling effort, uneven numbers of fish were caught across seasons, leading to data being combined across two years, with the wet season of 2019–2020 significantly drier compared to the 2020–2021 wet season^13^. Despite this, strong spatial structure was found, although we were unable to assess whether the strength of variation between seasons within a year would be stronger than the potential effect of variation of seasons between years. Additionally, the biology and ecology of the parasites of *P. diacanthus* remains largely unknown. Subsequently, we can only speculate on the life span of parasites and the possible influence of environmental variation on their abundance within a host population and their usefulness in assisting with host population structuring.

Of the parasitic taxa collected for the present study, not all were able to be identified to the lowest taxonomic unit possible. Despite a number of parasites not being described to the species level, we are confident that all taxa utilised in the analyses of this study are individual species. From the perspective of stock discrimination studies, the key to reliable analysis is not in the identification of a parasitic organism, but rather in the distinction of parasitic organisms^28^. Parasites are notoriously hard to identify and, although finer-scale differentiation of parasites would potentially increase inferential power^28^, the lack of species-level identification for some taxa did not prevent the identification of statistically significant parasite assemblage structure in this study.

## Conclusions

The utilisation of parasites as biological tags is well established^1,2,4,12,29,30^, and there is an increasing awareness of the need to consider potential impacts of spatial and temporal variability in biological and ecological parameters when studying a population and discriminating between stocks. The present study used parasites to confirm high levels of local variability for populations of *P. diacanthus* off northern Australia. Despite the temporal variation, parasite assemblages of *P. diacanthus* revealed strong spatial variation among locations within the studied regions, suggesting regional-scale population structure, and further indicating that adult fishes have limited connectivity and may remain resident in defined regions during the adult phase. The four sampled populations of *P. diacanthus* are clearly delineated by parasite assemblage, however, there may be some mixing of these populations during the tropical wet season. It is evident that temporal variability could be a confounding factor in the identification of nearshore stocks. Local environmental factors, such as increased monsoonal activity and freshwater influxes during the wet season period may alter fish behaviour and affect the survival and transmission success of parasitic organisms, ultimately varying host burdens and overall assemblage. Aligning with previous population structure research on *P. diacanthus,* our results suggest that *P. diacanthus* populations exhibit complex spatial structure over relatively small spatial scales and, thus, may be vulnerable to localised depletion and require appropriately scaled spatial management.

## Supplementary Information

Supplementary Information.

## Data Availability

All data produced for this study are provided in the manuscript. Supplementary material and raw data is available on the online version (Supplemental Materials).
