# Supplementary material for: Relationships between local variability in parasite communities of the black-spotted croaker (Protonibea diacanthus) (Teleostei: Sciaenidae) and host population structure and seasonality
Source: Sci Rep. 2023 Jun 25;13:10291. doi: 10.1038/s41598-023-37428-y (PMC10290980; doi:10.1038/s41598-023-37428-y)

**Supplementary Material**

**Relationships between local variability in parasite communities of the black-spotted croaker *(Protonibea diacanthus)* (Teleostei: Sciaenidae) and host population structure and seasonality**

Megan Porter* ^A, B^, Diane P. Barton ^A, B^, Mark Hearnden ^C^, Jo Randall ^C, D, E^, David A. Crook ^D, F^ and Shokoofeh Shamsi ^A, B^

^A^ School of Agricultural, Environmental and Veterinary Sciences, Charles Sturt University, Wagga Wagga, NSW 2678, Australia.

^B^ Gulbali Institute, Charles Sturt University, Wagga Wagga, NSW 2678, Australia

^C^ Department of Industry, Tourism and Trade, Northern Territory Government of Australia, Darwin, NT 0801, Australia.

^D^ Research Institute for the Environment and Livelihoods, Charles Darwin University, Casuarina, NT 0810, Australia.

^E^ Australian Institute of Marine Science, Arafura Timor Research Facility, Casuarina, NT 0810, Australia.

^F^ Department of Primary Industries, Narrandera Fisheries Centre, Narrandera, NSW 2700, Australia.

* Corresponding author. Email: mporter@csu.edu.au

|  |  | **Mid Dry** | | | | | | | |  | **Build Up** | | | | | | | |  | **Late Wet** | | | | | | | |
| --- | --- | --- | --- | --- | --- | --- | --- | --- | --- | --- | --- | --- | --- | --- | --- | --- | --- | --- | --- | --- | --- | --- | --- | --- | --- | --- | --- |
|  |  | **Caution Point** | | **Mitchell Point** | | **Sampan Creek** | | **Peron Islands** | |  | **Caution Point** | | **Mitchell Point** | | **Sampan Creek** | | **Peron Islands** | |  | **Caution Point** | | **Mitchell Point** | | **Sampan Creek** | | **Peron Islands** | |
|  | **No. fish sampled** | **9** | | **1** | | **8** | | **8** | |  | **10** | | **10** | | **10** | | **10** | |  | **8** | | **10** | | **5** | | **12** | |
|  | **Parasite taxa** | ***P* (%)** | ***A_M_*** | ***P* (%)** | ***A_M_*** | ***P* (%)** | ***A_M_*** | ***P* (%)** | ***A_M_*** |  | ***P* (%)** | ***A_M_*** | ***P* (%)** | ***A_M_*** | ***P* (%)** | ***A_M_*** | ***P* (%)** | ***A_M_*** |  | ***P* (%)** | ***A_M_*** | ***P* (%)** | ***A_M_*** | ***P* (%)** | ***A_M_*** | ***P* (%)** | ***A_M_*** |
| **Ectoparasites** | **Isopod** | 11.11 | 0.11 | 0.00 | 0.00 | 0.00 | 0.00 | 0.00 | 0.00 |  | 0.00 | 0.00 | 0.00 | 0.00 | 0.00 | 0.00 | 0.00 | 0.00 |  | 0.00 | 0.00 | 0.00 | 0.00 | 0.00 | 0.00 | 0.00 | 0.00 |
|  | ***Caligus* sp.** | 22.22 | 0.22 | 0.00 | 0.00 | 25.00 | 0.50 | 75.00 | 2.13 |  | 10.00 | 0.10 | 0.00 | 0.00 | 10.00 | 0.10 | 0.00 | 0.00 |  | 0.00 | 0.00 | 0.00 | 0.00 | 20.00 | 0.20 | 33.33 | 0.67 |
|  | ***Lernanthropus paracruciatus*** | 100.00 | 2.00 | 100.00 | 1.00 | 100.00 | 5.00 | 100.00 | 5.38 |  | 90.00 | 4.40 | 90.00 | 6.00 | 100.00 | 19.10 | 90.00 | 4.70 |  | 87.50 | 6.50 | 70.00 | 1.90 | 20.00 | 1.20 | 100.00 | 2.67 |
|  | **Diplectanidae** | 100.00 | 197.78 | 100.00 | 120.00 | 100.00 | 840.25 | 100.00 | 504.00 |  | 100.00 | 155.70 | 100.00 | 159.10 | 100.00 | 359.10 | 100.00 | 187.10 |  | 100.00 | 2210.00 | 100.00 | 313.00 | 100.00 | 1102.00 | 100.00 | 951.67 |
| **Endoparasites** | ***Poecilacanstrium* sp.** | 100.00 | 18.33 | 100.00 | 4.00 | 100.00 | 61.88 | 100.00 | 39.63 |  | 100.00 | 37.20 | 90.00 | 17.00 | 100.00 | 37.70 | 100.00 | 51.30 |  | 100.00 | 24.00 | 100.00 | 26.40 | 100.00 | 45.40 | 100.00 | 25.33 |
|  | **Digenean metacercariae** | 0.00 | 0.00 | 0.00 | 0.00 | 87.50 | 4.00 | 0.00 | 0.00 |  | 10.00 | 0.10 | 10.00 | 0.10 | 10.00 | 0.30 | 0.00 | 0.00 |  | 50.00 | 0.63 | 10.00 | 0.20 | 60.00 | 2.20 | 25.00 | 0.25 |
|  | **Cucullanidae** | 100.00 | 23.44 | 100.00 | 7.00 | 87.50 | 32.38 | 87.50 | 19.50 |  | 80.00 | 25.00 | 100.00 | 18.90 | 90.00 | 19.80 | 90.00 | 8.00 |  | 100.00 | 70.88 | 100.00 | 12.90 | 100.00 | 23.60 | 100.00 | 13.00 |
|  | **Anisakidae** | 0.00 | 0.00 | 0.00 | 0.00 | 50.00 | 0.75 | 0.00 | 0.00 |  | 0.00 | 0.00 | 10.00 | 0.10 | 0.00 | 0.00 | 0.00 | 0.00 |  | 0.00 | 0.00 | 0.00 | 0.00 | 0.00 | 0.00 | 0.00 | 0.00 |
|  | **Other nematodes** | 55.56 | 0.56 | 0.00 | 0.00 | 0.00 | 0.00 | 25.00 | 0.50 |  | 0.00 | 0.00 | 10.00 | 0.10 | 30.00 | 0.40 | 40.00 | 0.40 |  | 0.00 | 0.00 | 0.00 | 0.00 | 60.00 | 1.20 | 16.67 | 0.42 |
|  | ***Orientodiploproctodaeum* sp.** | 100.00 | 28.78 | 0.00 | 0.00 | 87.50 | 11.38 | 100.00 | 52.50 |  | 100.00 | 49.30 | 50.00 | 13.90 | 90.00 | 15.80 | 100.00 | 26.30 |  | 100.00 | 38.75 | 100.00 | 18.50 | 100.00 | 5.20 | 91.67 | 23.17 |
|  | ***Stephanostomum* sp.** | 88.89 | 10.89 | 100.00 | 6.00 | 100.00 | 57.38 | 75.00 | 9.13 |  | 100.00 | 21.80 | 90.00 | 28.80 | 100.00 | 14.90 | 90.00 | 6.00 |  | 87.50 | 35.50 | 100.00 | 17.30 | 90.00 | 17.40 | 100.00 | 5.75 |
|  | ***Pleorchis* sp.** | 0.00 | 0.00 | 0.00 | 0.00 | 0.00 | 0.00 | 12.50 | 0.25 |  | 30.00 | 0.80 | 60.00 | 6.60 | 0.00 | 0.00 | 40.00 | 0.60 |  | 12.50 | 0.25 | 40.00 | 0.70 | 0.00 | 0.00 | 25.00 | 0.42 |
|  | **Hemiuridae** | 0.00 | 0.00 | 0.00 | 0.00 | 12.50 | 0.13 | 37.50 | 1.00 |  | 0.00 | 0.00 | 0.00 | 0.00 | 0.00 | 0.00 | 0.00 | 0.00 |  | 12.50 | 0.38 | 10.00 | 0.20 | 20.00 | 0.20 | 33.33 | 0.50 |
|  | **Opecoelidae** | 11.11 | 0.11 | 0.00 | 0.00 | 50.00 | 0.63 | 75.00 | 1.75 |  | 10.00 | 0.10 | 40.00 | 0.40 | 10.00 | 0.20 | 20.00 | 0.20 |  | 0.00 | 0.00 | 0.00 | 0.00 | 40.00 | 0.40 | 33.33 | 0.83 |
|  | **Unidentified Digenean** | 0.00 | 0.00 | 0.00 | 0.00 | 0.00 | 0.00 | 0.00 | 0.00 |  | 10.00 | 0.10 | 0.00 | 0.00 | 0.00 | 0.00 | 0.00 | 0.00 |  | 0.00 | 0.00 | 0.00 | 0.00 | 0.00 | 0.00 | 0.00 | 0.00 |

**Table S1a: Prevalence and mean abundance of parasites from Protonibea diacanthus in year 1 (2019-2020), across different nearshore and offshore sites of the Northern Territory, from different seasons of collection. A_M_, mean abundance; P, prevalence of infection, expressed as a percentage.**

**Table S1b: Prevalence and mean abundance of parasites from Protonibea diacanthus in year 2 (2020-2021), across different nearshore and offshore sites of the Northern Territory, from different seasons of collection. A_M_, mean abundance; P, prevalence of infection, expressed as a percentage.**

|  |  | **Mid Dry** | | | | | | | |  | **Build Up** | | | | | | | |  | **Late Wet** | | | | | | | |
| --- | --- | --- | --- | --- | --- | --- | --- | --- | --- | --- | --- | --- | --- | --- | --- | --- | --- | --- | --- | --- | --- | --- | --- | --- | --- | --- | --- |
|  |  | **Caution Point** | | **Mitchell Point** | | **Sampan Creek** | | **Peron Islands** | |  | **Caution Point** | | **Mitchell Point** | | **Sampan Creek** | | **Peron Islands** | |  | **Caution Point** | | **Mitchell Point** | | **Sampan Creek** | | **Peron Islands** | |
|  | **No. fish sampled** | **10** | | **10** | | **12** | | **19** | |  | **7** | | **10** | | **10** | | **10** | |  | **8** | | **10** | | **9** | | **10** | |
|  | **Parasite taxa** | ***P* (%)** | ***A_M_*** | ***P* (%)** | ***A_M_*** | ***P* (%)** | ***A_M_*** | ***P* (%)** | ***A_M_*** |  | ***P* (%)** | ***A_M_*** | ***P* (%)** | ***A_M_*** | ***P* (%)** | ***A_M_*** | ***P* (%)** | ***A_M_*** |  | ***P* (%)** | ***A_M_*** | ***P* (%)** | ***A_M_*** | ***P* (%)** | ***A_M_*** | ***P* (%)** | ***A_M_*** |
| **Ectoparasites** | **Isopod** | 0.00 | 0.00 | 0.00 | 0.00 | 0.00 | 0.00 | 0.00 | 0.00 |  | 0.00 | 0.00 | 10.00 | 0.10 | 0.00 | 0.00 | 0.00 | 0.00 |  | 0.00 | 0.00 | 0.00 | 0.00 | 11.11 | 0.11 | 0.00 | 0.00 |
|  | ***Caligus* sp.** | 0.00 | 0.00 | 0.00 | 0.00 | 16.67 | 0.25 | 63.16 | 1.32 |  | 28.57 | 0.29 | 0.00 | 0.00 | 0.00 | 0.00 | 20.00 | 0.20 |  | 0.00 | 0.00 | 0.00 | 0.00 | 0.00 | 0.00 | 10.00 | 0.10 |
|  | ***Lernanthropus paracruciatus*** | 60.00 | 2.10 | 100.00 | 6.70 | 75.00 | 2.50 | 36.84 | 1.37 |  | 71.43 | 3.14 | 100.00 | 5.70 | 90.00 | 6.70 | 100.00 | 4.20 |  | 100.00 | 9.38 | 70.00 | 3.30 | 100.00 | 8.44 | 80.00 | 2.40 |
|  | **Diplectanidae** | 100.00 | 1406.70 | 100.00 | 1223.00 | 100.00 | 946.92 | 100.00 | 2028.42 |  | 100.00 | 1262.71 | 100.00 | 2498.00 | 100.00 | 31.20 | 100.00 | 39.60 |  | 100.00 | 3107.50 | 100.00 | 1345.00 | 100.00 | 3293.33 | 100.00 | 26.00 |
| **Endoparasites** | ***Poecilacanstrium* sp.** | 100.00 | 35.60 | 100.00 | 21.50 | 100.00 | 40.17 | 100.00 | 34.68 |  | 100.00 | 16.14 | 100.00 | 25.70 | 100.00 | 40.40 | 100.00 | 29.60 |  | 100.00 | 28.50 | 100.00 | 19.40 | 100.00 | 46.56 | 90.00 | 12.80 |
|  | **Digenean metacercariae** | 20.00 | 0.30 | 0.00 | 0.00 | 41.67 | 0.67 | 10.53 | 0.26 |  | 0.00 | 0.00 | 0.00 | 0.00 | 0.00 | 0.00 | 40.00 | 1.30 |  | 0.00 | 0.00 | 0.00 | 0.00 | 33.33 | 0.44 | 20.00 | 0.20 |
|  | **Cucullanidae** | 100.00 | 20.50 | 90.00 | 24.40 | 100.00 | 25.50 | 100.00 | 14.32 |  | 100.00 | 18.86 | 100.00 | 11.80 | 80.00 | 28.20 | 100.00 | 10.70 |  | 87.50 | 42.00 | 100.00 | 13.00 | 100.00 | 57.89 | 100.00 | 6.00 |
|  | **Anisakidae** | 10.00 | 0.10 | 0.00 | 0.00 | 8.33 | 0.08 | 0.00 | 0.00 |  | 0.00 | 0.00 | 0.00 | 0.00 | 30.00 | 0.60 | 10.00 | 0.20 |  | 0.00 | 0.00 | 0.00 | 0.00 | 0.00 | 0.00 | 0.00 | 0.00 |
|  | **Other nematodes** | 0.00 | 0.00 | 10.00 | 0.10 | 16.67 | 0.17 | 31.58 | 0.63 |  | 0.00 | 0.00 | 10.00 | 0.30 | 10.00 | 0.10 | 10.00 | 0.10 |  | 0.00 | 0.00 | 0.00 | 0.00 | 0.00 | 0.00 | 10.00 | 0.20 |
|  | ***Orientodiploproctodaeum* sp.** | 100.00 | 15.00 | 100.00 | 39.10 | 83.33 | 4.00 | 100.00 | 40.05 |  | 100.00 | 19.43 | 100.00 | 38.30 | 80.00 | 10.30 | 100.00 | 26.40 |  | 100.00 | 25.75 | 80.00 | 9.00 | 88.88 | 19.33 | 100.00 | 25.80 |
|  | ***Stephanostomum* sp.** | 90.00 | 18.30 | 100.00 | 33.30 | 75.00 | 8.08 | 89.47 | 11.42 |  | 100.00 | 12.86 | 100.00 | 21.00 | 70.00 | 5.50 | 70.00 | 4.90 |  | 75.00 | 26.25 | 70.00 | 5.30 | 100.00 | 57.00 | 30.00 | 0.60 |
|  | ***Pleorchis* sp.** | 20.00 | 0.20 | 80.00 | 5.30 | 0.00 | 0.00 | 5.26 | 0.21 |  | 0.00 | 0.00 | 30.00 | 0.40 | 0.00 | 0.00 | 0.00 | 0.00 |  | 12.50 | 0.25 | 10.00 | 0.30 | 0.00 | 0.00 | 0.00 | 0.00 |
|  | **Hemiuridae** | 0.00 | 0.00 | 0.00 | 0.00 | 25.00 | 0.25 | 10.53 | 0.11 |  | 14.29 | 0.14 | 0.00 | 0.00 | 0.00 | 0.00 | 0.00 | 0.00 |  | 0.00 | 0.00 | 10.00 | 0.10 | 12.50 | 0.78 | 20.00 | 0.30 |
|  | **Opecoelidae** | 0.00 | 0.00 | 0.00 | 0.00 | 8.33 | 0.17 | 0.00 | 0.00 |  | 0.00 | 0.00 | 10.00 | 0.20 | 0.00 | 0.00 | 0.00 | 0.00 |  | 0.00 | 0.00 | 0.00 | 0.00 | 0.00 | 0.00 | 10.00 | 0.10 |
|  | **Unidentified Digenean** | 0.00 | 0.00 | 0.00 | 0.00 | 0.00 | 0.00 | 0.00 | 0.00 |  | 0.00 | 0.00 | 0.00 | 0.00 | 0.00 | 0.00 | 0.00 | 0.00 |  | 0.00 | 0.00 | 0.00 | 0.00 | 0.00 | 0.00 | 0.00 | 0.00 |

**Table S2: Percentage of reclassification from all locations with seasons combined**

|  | CP | MP | PI | SC |
| --- | --- | --- | --- | --- |
| CP | 48.08 | 15.38 | 26.92 | 9.62 |
| MP | 34.00 | 40.00 | 8.00 | 18.00 |
| PI | 18.84 | 5.80 | 60.87 | 14.49 |
| SC | 15.79 | 1.75 | 17.54 | 64.91 |

Note: Percentage successfully reclassified = 54%, Proportion chance criterion = 25%

**Table S3: Percentage of reclassification from all locations during mid-dry**

|  | CP-M | MP-M | PI-M | SC-M |
| --- | --- | --- | --- | --- |
| CP-M | 57.89 | 5.26 | 26.32 | 10.53 |
| MP-M | 10.00 | 80.00 | 0.00 | 10.00 |
| PI-M | 14.82 | 3.70 | 77.78 | 3.70 |
| SC-M | 17.39 | 0.00 | 13.04 | 69.57 |

Note: Percentage successfully reclassified = 71%, Proportion chance criterion = 28%

**Table S4: Percentage of reclassification from all locations during build-up**

|  | CP-B | MP-B | PI-B | SC-B |
| --- | --- | --- | --- | --- |
| CP-B | 58.82 | 17.65 | 17.65 | 5.88 |
| MP-B | 25.00 | 50.00 | 10.00 | 15.00 |
| PI-B | 35.00 | 5.00 | 35.00 | 25.00 |
| SC-B | 10.00 | 5.00 | 35.00 | 50.00 |

Note: Percentage successfully reclassified = 48%, Proportion chance criterion = 25%

**Table S5: Percentage of reclassification from all locations during late-wet**

|  | CP-L | MP-L | PI-L | SC-L |  |
| --- | --- | --- | --- | --- | --- |
| CP-L | 37.50 | 37.50 | 12.50 | 12.50 |  |
| MP-L | 15.00 | 55.00 | 5.00 | 25.00 |  |
| PI-L | 18.18 | 13.64 | 68.18 | 0.00 |  |
| SC-L | 28.57 | 21.43 | 14.29 | 35.71 |  |

Note: Percentage successfully reclassified = 51%, Proportion chance criterion = 26%

**Table S6: Percentage of reclassification from all seasons at Caution Point**

|  | CP-B | CP-L | CP-M |
| --- | --- | --- | --- |
| CP-B | 64.71 | 23.53 | 11.76 |
| CP-L | 6.25 | 75.00 | 18.75 |
| CP-M | 26.32 | 31.58 | 42.10 |

Note: Percentage successfully reclassified = 60%, Proportion chance criterion = 34%

**Table S7: Percentage of reclassification from all seasons at Mitchell Point**

|  | MP-B | MP-L | MP-M |
| --- | --- | --- | --- |
| MP-B | 40.00 | 55.00 | 5.00 |
| MP-L | 25.00 | 70.00 | 5.00 |
| MP-M | 30.00 | 20.00 | 50.00 |

Note: Percentage successfully reclassified = 54%, Proportion chance criterion = 36%

**Table S8: Percentage of reclassification from all seasons at Sampan Creek**

|  | SC-B | SC-L | SC-M |
| --- | --- | --- | --- |
| SC-B | 90.00 | 0.00 | 10.00 |
| SC-L | 7.14 | 14.29 | 78.57 |
| SC-M | 0.00 | 30.43 | 69.57 |

Note: Percentage successfully reclassified = 63%, Proportion chance criterion = 35%

**Table S9: Percentage of reclassification from all seasons at Peron Islands**

|  | PI-B | PI-L | PI-M |
| --- | --- | --- | --- |
| PI-B | 80.00 | 20.00 | 0.00 |
| PI-L | 22.73 | 36.36 | 40.91 |
| PI-M | 3.70 | 29.63 | 66.67 |

Note: Percentage successfully reclassified = 61%, Proportion chance criterion = 34%

**Table S10: Percentage of reclassification from all locations and seasons**

|  | CP-B | CP-L | CP-M | MP-B | MP-L | MP-M | PI-B | PI-L | PI-M | SC-B | SC-L | SC-M |
| --- | --- | --- | --- | --- | --- | --- | --- | --- | --- | --- | --- | --- |
| CP-B | 17.65 | 17.65 | 11.76 | 5.88 | 5.88 | 5.88 | 23.53 | 0.00 | 5.88 | 5.88 | 0.00 | 0.00 |
| CP-L | 6.25 | 37.50 | 0.00 | 6.25 | 12.50 | 12.50 | 0.00 | 12.50 | 0.00 | 0.00 | 12.50 | 0.00 |
| CP-M | 5.26 | 10.53 | 10.53 | 0.00 | 31.58 | 0.00 | 0.00 | 15.79 | 10.53 | 10.53 | 0.00 | 5.26 |
| MP-B | 0.00 | 20.00 | 0.00 | 10.00 | 15.00 | 25.00 | 5.00 | 5.00 | 0.00 | 15.00 | 5.00 | 0.00 |
| MP-L | 15.00 | 10.00 | 10.00 | 10.00 | 30.00 | 5.00 | 0.00 | 5.00 | 0.00 | 5.00 | 5.00 | 5.00 |
| MP-M | 0.00 | 10.00 | 0.00 | 10.00 | 10.00 | 70.00 | 0.00 | 0.00 | 0.00 | 0.00 | 0.00 | 0.00 |
| PI-B | 20.00 | 0.00 | 5.00 | 5.00 | 10.00 | 0.00 | 35.00 | 5.00 | 0.00 | 10.00 | 0.00 | 10.00 |
| PI-L | 0.00 | 13.64 | 4.55 | 9.09 | 9.09 | 0.00 | 9.09 | 13.64 | 27.27 | 13.64 | 0.00 | 0.00 |
| PI-M | 3.70 | 3.70 | 3.70 | 0.00 | 7.41 | 3.70 | 0.00 | 22.22 | 51.85 | 0.00 | 3.70 | 0.00 |
| SC-B | 10.00 | 0.00 | 5.00 | 5.00 | 0.00 | 0.00 | 20.00 | 0.00 | 0.00 | 55.00 | 0.00 | 5.00 |
| SC-L | 7.14 | 21.43 | 0.00 | 0.00 | 14.29 | 0.00 | 0.00 | 0.00 | 7.14 | 0.00 | 14.29 | 35.71 |
| SC-M | 0.00 | 0.00 | 4.35 | 0.00 | 17.39 | 0.00 | 4.35 | 8.70 | 8.70 | 0.00 | 8.70 | 47.83 |

Note: Percentage successfully reclassified = 32%, Proportion chance criterion = 9%

**Fig S1:** Linear discriminant function analysis (LDFA) of parasite assemblages of all fish from all locations and seasons.

**Legend**: B, build-up; L, late-wet; M, mid-dry; CP, Caution Point; MP, Mitchell Point; PI, Peron Islands; SC, Sampan Creek


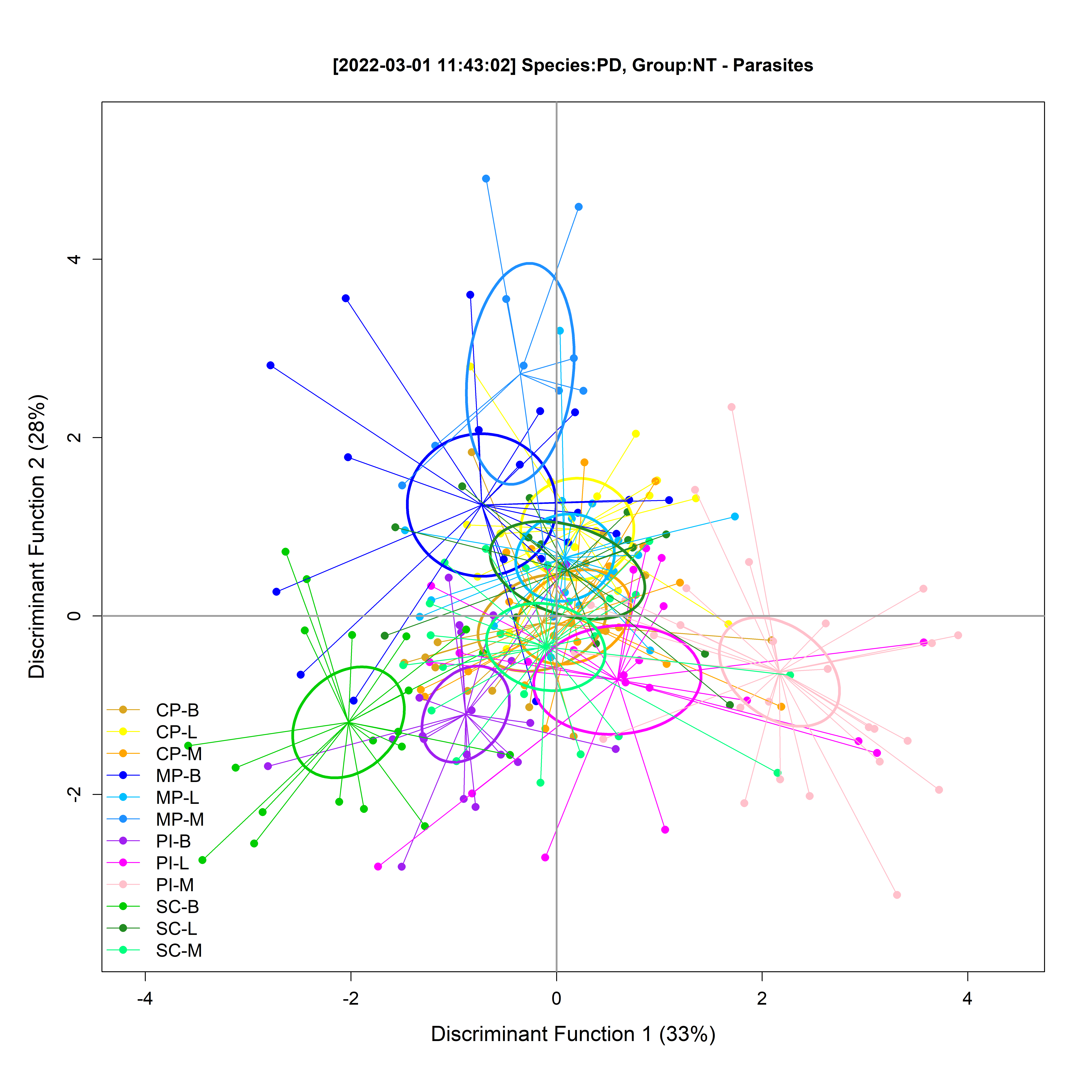

Supplement: Supplementary file 1 — Supplementary Information. [file 41598_2023_37428_MOESM1_ESM.docx]
